# Practical pathological methods for reliable diagnosis of secretory carcinomas of the salivary gland

**DOI:** 10.1007/s12672-025-03072-3

**Published:** 2025-07-03

**Authors:** Ryo Kawaura, Tomohiko Ishikawa, Hirofumi Shibata, Masashi Kuroki, Kazuhiro Kobayashi, Yuki Hanamatsu, Tatsuhiko Miyazaki, Hiroyuki Tomita, Takuma Ishihara, Hajime Usubuchi, Yayoi Aoyama, Yukinori Asada, Takayuki Imai, Akira Ohkoshi, Akira Hara, Yukio Katori, Toru Furukawa, Takenori Ogawa

**Affiliations:** 1 Department of Otolaryngology-Head and Neck Surgery, Gifu University Graduate School of Medicine, 1-1 Yanagido, Gifu, 501-1194 Japan; 2 Department of Otolaryngology-Head and Neck Surgery, Tohoku University Graduate School of Medicine, 1-1 Seiryo-machi, Aoba-ku, Sendai, 980-8575 Miyagi Japan; 3 Department of Pathology, Tohoku University Graduate School of Medicine, 1-1 Seiryo-machi, Aoba-ku, Sendai, 980-8575 Miyagi Japan; 4 Department of Tumor Pathology, Gifu University Graduate School of Medicine, 1-1 Yanagido, Gifu, 501-1194 Japan; 5 Department of Pathology, Gifu University Hospital, 1-1 Yanagido, Gifu, 501- 1194 Japan; 6 Center for One Medicine Innovative Translational Research, Gifu University Institute for Advanced Study, 1-1 Yanagido, Gifu, 501-1194 Japan; 7 Innovative and Clinical Research Promotion Center, Gifu University Hospital, 1-1 Yanagido, Gifu, 501-1194 Japan; 8 Department of Pathology, Sendai Kousei Hospital, 1-20 Tsutsumidori Amamiya-machi, Aoba-ku, Sendai, 981-0914 Miyagi Japan; 9 Department of Investigative Pathology, Tohoku University Hospital, 1-1 Seiryo-machi, Aoba-ku, Sendai, 980-8575 Miyagi Japan; 10 Department of Head and Neck Surgery, Miyagi Cancer Center, 47-1 Medeshima, Natori, 981-1293 Miyagi Japan

**Keywords:** Salivary gland, Secretory carcinoma, Acinic cell carcinoma, *ETV6:NTRK3* fusion gene, S-100, Mammaglobin

## Abstract

Secretory carcinomas (SCs) of the salivary gland have recently been recognized as low-grade, malignant tumors. Before this designation, most SCs were diagnosed as variants of acinic cell carcinomas (AciCCs). SCs harbor the t(12;15)(p13;q25) translocation that generates an oncogenic fusion gene, *ETS variant transcription factor 6/Neurotrophic tyrosine receptor kinase(ETV6::NTRK3)*. However, detecting fusion genes in a clinical setting is time-consuming and costly. In this study, we examined 31 cases previously diagnosed as AciCC and SC using pathological analyses with detection of fusion genes using *ETV6* break-apart fluorescence in-situ hybridization and reverse transcription-polymerase chain reaction. After re-analysis, we found that these 31 cases actually comprised 21 SCs and 10 AciCCs. We examined the diagnostic utility of immunohistochemistry by comparing results with the fusion gene, Pan-Trk, which despite having recently been reported as effective for diagnosis of SC, was not universally accurate. However, combining mammaglobin and S-100 could be particularly useful in diagnosing SC. This practical method will contribute to accurate diagnosis of SCs, while saving time in daily clinical practice.

**Supplementary Information:**

The online version contains supplementary material available at 10.1007/s12672-025-03072-3.

## Introduction

Secretory carcinomas (SC) is salivary gland cancer recognized in 2010 [1]. Before this definition, most cases of SC were probably diagnosed as papillary-cystic, microcystic, or follicular types of acinic cell carcinoma (AciCC) [1, 2]. Skalova et al. (2010) first proposed a new subclassification of salivary gland cancer, called mammary analog secretory carcinoma (MASC). MASC was defined by its histological similarity to mammary gland SC, with which it shares the t(12;15)(p13;q25) translocation. In the 2017 WHO classification, SC was coined as the term for MASC in the salivary gland [3]. Since that initial report, various studies have detailed the tumorigenic nature of SC, including its clinical course, histological characteristics, and molecular features. In general, both SC and AciCC are classified as low-grade salivary gland carcinomas. However, before the establishment of SC [4], some cases of SC [5] and AciCC showed high-grade transformation (HGT). In terms of its genetic profile, the *ETS variant transcription factor 6/Neurotrophic tyrosine receptor kinase (ETV6::NTRK3)* gene fusion was initially considered a characteristic feature of SC in salivary glands; however, alternative gene fusions, such as *ETV6::RET* [6] and *ETV6::MAML3* [7], have also been reported. Recently, anticancer drugs for *NTRK* fusion-positive solid tumors, entrectinib [8] and larotrectinib [9] have become available [10]. In addition, the LIBRETTO-001 trial in patients with *RET* fusion gene-positive advanced or metastatic solid tumors benefit from selpercatinib [11]. These results strongly suggest that accurate differentiation between SC and AciCC may determine post-operative treatment strategies and improve subsequent patient prognosis [3, 12].

However, due to constraints such as time and cost, gene analysis cannot always be performed in clinical practice. Effective molecular profiling is required, but this is not a method that can be performed quickly and easily, like IHC. Accordingly, more efficient methods are needed to distinguish SC and AciCC.

Diagnosing SC and AciCC in typical cases using histology and immunohistochemistry (IHC) without molecular profiling (FISH or RT-PCR) is not very difficult. Histologically, typical AciCC is composed of zymogen granule-rich, well-differentiated cells that form a solid or micro-cystic pattern [13]. In contrast, typical SC comprises eosinophilic/clear cells forming papillary-cystic, micro-cystic, or follicular tumors with abundant eosinophilic secretions. Regarding IHC, SC is more likely to be positive for S-100, vimentin and mammaglobin, whereas many cases of AciCC are positive for DOG1 staining [13]. However, in atypical cases, both may exhibit similar pathological traits. Recent reports have shown that some SCs are present in cases diagnosed pathologically as AciCC [1].

In this study, we first confirmed the existence of fusion genes in 31 cases previously diagnosed pathologically as AciCC/SC and then made a definitive diagnosis. Based on these results, we sought to establish an effective IHC combination to accurately distinguish SC from AciCC using immunohistochemistry commonly employed in clinical practice.

## Materials and methods

### Patients

We reviewed 31 cases of salivary gland tumors originally diagnosed as AciCC or SC with available clinicopathological information and histological specimens. Cases aged 20 years or older were included, included consecutively. Patients included 15 males/16 females, with a mean age of 55.1 years, and median age of 53 years (range 25–85 years). They comprised 27 parotid gland cases, 1 submandibular gland case, and 3 minor salivary gland cases, including 18 cases at Tohoku University and Miyagi Cancer Center from 1993 to 2018 and 14 cases at Gifu University, Gifu Prefectural General Medical Center, and Ogaki Municipal Hospital from 2004 to 2023. Patients are included consecutively during this period. TNM staging was made following the Union for International Cancer Control (UICC) 8th edition. This study was approved by the ethics committees of Tohoku University Graduate School of Medicine (#2019-1-082) and Gifu University Graduate School of Medicine (#2022 − 124).

### FISH detection of *ETV6* gene rearrangements

FISH was performed on unstained, 4-µm sections of FFPE using a Vysis LSI ETV6 Dual Color, Break Apart Rearrangement Probe (Abbott Molecular, Chicago, IL) according to the manufacturer’s instructions. Briefly, deparaffinization, heat treatment, proteolysis, denaturation, hybridization, and washing were performed. The cutoff value for the *ETV6* split was 10%.

### Detection of *ETV6-NTRK3* and *ETV6-RET* fusion transcripts by RT-PCR

RNA was extracted from formalin-fixed, paraffin-embedded (FFPE) tissues using RecoverAll Total Nucleic Acid Isolation Kits for FFPE (Ambion, Austin, TX), and cDNA was synthesized with PrimeScript RT reagent Kits (Takara Bio, Shiga, Japan). RNA quality was checked using a Nanodrop (Thermo Fisher Scientific), with threshold values of A260/280: 1.8 and A260/230: 1.6. All procedures were performed according to the manufacturer’s instructions. RT-PCR was performed using a published method [14]. Primers used are listed in Supplementary Table 1. Detection of fusion transcripts (exon 5-exon 15, exon 4-exon 14, exon 5-exon 14 of *ETV6::NTRK3*, and exon 6-exon 12 of *ETV6::RET*) was performed using RT-PCR. Nested RT-PCR was performed following Skalova et al., 2016 [14], as follows: For primary PCR, 1 µL cDNA was added to a reaction consisting of 10.0 µL AmpliTaq Gold 360 Master Mix (Applied Biosystems, Foster City, CA), 10 pmol of each primer [14], and distilled water up to 25 µL. The amplification program comprised denaturation at 94 °C for 2 min, followed by 25 cycles of denaturation at 94 °C for 30 s, annealing at 55–60 °C (based on the temperature determined in the previous study [14]) for 30 s, and extension at 68 °C for 30 s. The program was completed by incubation at 68 °C for 5 min. For nested PCR, 2 µL of an amplified product of the first PCR was used as a template with primers designed for nested PCRs [14] using the same program as first-round PCR, except that 35 amplification cycles were used. Samples from SC cases in which the *ETV6::NTRK3* fusion gene had been identified by FISH analysis and comprehensive genome profiling were used as positive controls.

### DNA sequencing analysis

Fully amplified PCR products were treated using a QIAquick PCR Purification Kit (QIAGEN, Hilden, Germany) and analyzed with an ABI 3500xL Genetic Analyzer (Applied Biosystems). All procedures were performed according to manufacturer instructions. DNA sequences were used to detect fusion genes described above on consignment. Briefly, DNA sequence for chain termination PCR, size separation by gel electrophoresis, and gel analysis and determination of DNA sequence were conducted. Reference DNA sequences were used from the ensemble genome browser 111.

### Histological and immunohistochemical analysis

Hematoxylin and eosin (HE) and immunohistochemistry (IHC) were performed on 4-µm sections of FFPE. IHC employed the following primary antibodies: anti-S-100 (A103, 1:10 dilution, Biogenex, Fremont, CA), anti-DOG1 (Nichirei Bioscience, polyclonal, prediluted), anti-mammaglobin (EP249, 1: 2 dilution, Leica, Wetzlar, Germany), anti-GATA3 (L50-823, 1:500 dilution, Biocare Medical, Pacheco, CA), anti-Ki-67 (MIB-1, prediluted, Agilent Technologies), and anti-pan-Trk (EPR17341, 1:500 dilution, Abcam, Cambridge, UK). Histofine SAB-PO kit (Nichirei, Tokyo, Japan) was used for anti-DOG1, anti-pan-Trk, and anti-GATA3. Autostainer Link48 (Agilent Technologies) was used for anti-Ki-67. EnVision FLEX (Agilent Technologies) was used for anti-S-100 and anti-mammaglobin. S100 was stained in the nucleus, cytoplasm, and cell membrane. Similarly, mammaglobin was stained in the cytoplasm, GATA3 in the nucleus and cytoplasm, Ki67 in the nucleus, DOG1 in the cytoplasm and cell membrane, and pan-Trk in the nucleus and cytoplasm. More than 5% of stained cells were considered positive. For anti-pan-Trk, samples from SC cases in which the *ETV6::NTRK3* fusion gene had been identified by FISH analysis and comprehensive genome profiling were used as positive controls. For other antibodies, specimens from salivary gland cancers and tumors other than SC/AciCC that had been previously diagnosed and had undergone IHC were used as controls. Some samples were stained manually under the same conditions as machine staining. Automatic staining was performed first. In the manual staining performed later, samples that had already been automatically stained were also stained manually, and a pathologist evaluated them to confirm that staining was of the same level. In the manual staining, samples were soaked in xylene for 5 min three times to remove paraffin and 100% ethanol for 5 min three times to hydrophilize. After washing with water for several min, samples were soaked in Tris-EDTA (pH 9.0) or citrate (pH 6.0) buffer with the Decloaking Chamber NxGen (Biocare Medical, Pacheco, CA) for 10 min. By blocking endogenous peroxidase with 3% hydrogen peroxide solution and non-specific binding with 2% bovine serum albumin (BSA) in PBS, primary antibodies were applied at room temperature overnight. After washing with PBS, samples were processed with Histofine SAB-PO kit (Nichirei, Tokyo, Japan) as secondary antibodies for 30 min at room temperature. Samples were washed twice in PBS for 5 min, followed by color development using 3,3’-diaminobenzidine for 5–10 min. Sections were counterstained with hematoxylin, dehydrated in graded alcohol and xylene, and mounted. Histological features of all tumors and IHC were reviewed by pathologists (TI, HU, KK, and YH). Pathologists performed the IHC evaluation blinded to preliminary and final diagnoses.

### Statistical analysis

Patient characteristics were summarized by median and interquartile range (IQR) for continuous variables, and by frequency for categorical variables. Statistical tests were exploratory. The Wilcoxon rank sum test was used to identify differences between cases of SC and AciCC for continuous variables such as age and tumor size. Fisher’s exact test was used to determine differences in categorical data. Differences in the rate of overall survival and progression-free survival were estimated using the Kaplan-Meier method. Rate differences between SC and AciCC were tested using the log-rank test. The predicted probability of SC combined with IHC was obtained using a logistic regression model. Two factors, IHC and its interaction, were included in the logistic regression model. Two-sided p-values less than 0.05 were considered statistically significant. All statistical analyses were computed using R for Mac (4.3.2 GUI 1.80 Big Sur Intel build (8281)).

## Results

### FISH analysis detected rearrangements of the *ETV6* gene

**Fig. 1:**
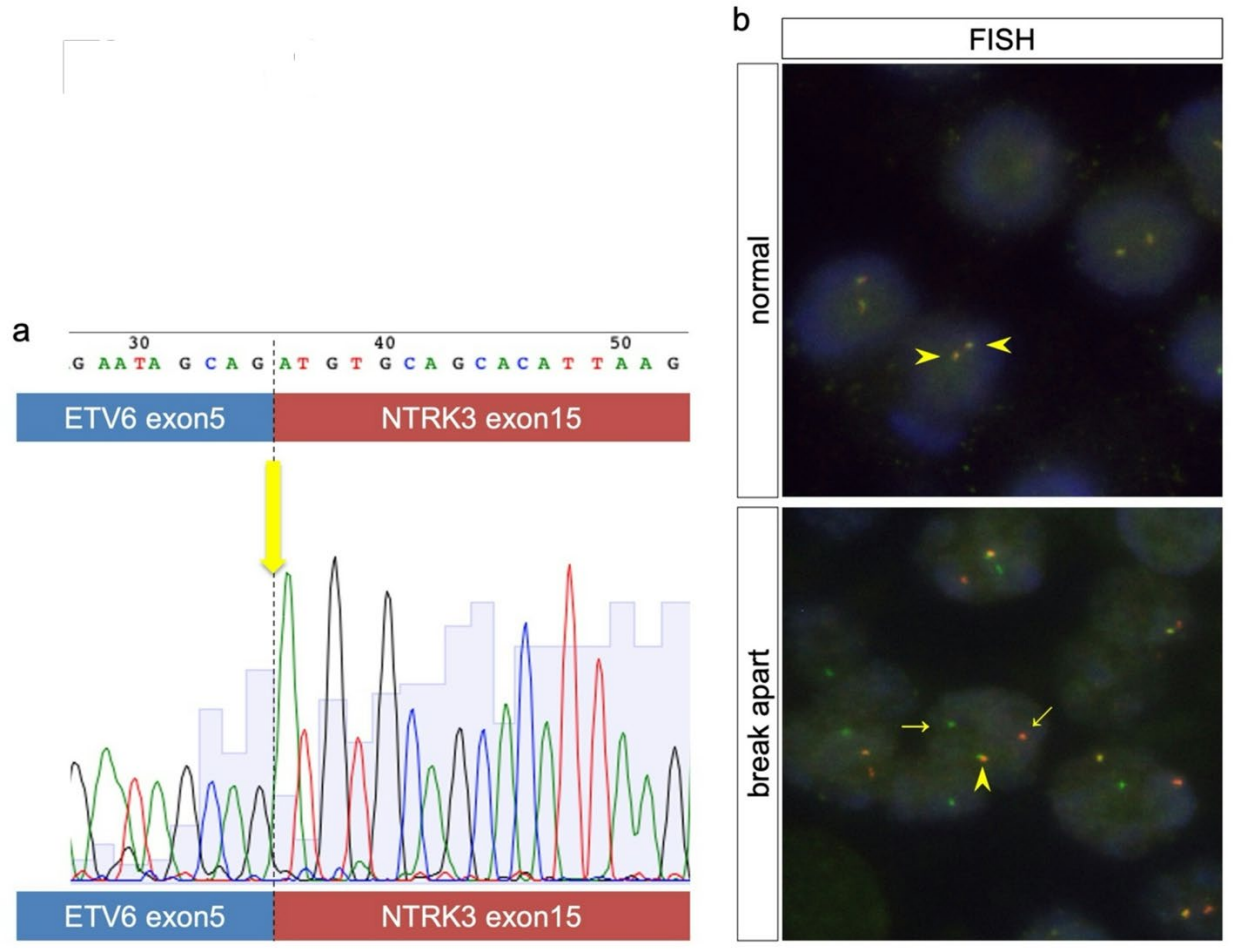
Analysis of fusion genes. (a) Sanger sequencing analysis. Sequencing analysis of an amplicon generated using *ETV6*::*NTRK3* fusion primers in patient G_#1(SC10). *ETV6* exon 5::*NTRK3* exon 15 in-frame fusion, which is the classical gene rearrangement of SC, was also observed in SC patients. (b) *ETV6*-FISH analysis. Normal pattern signals show two yellow signals (arrowheads) because of close green and red signals indicating *ETV6* without a break in each nucleus. Typical break-apart pattern signals show isolated green and red signals (arrows) indicating a break in *ETV6* and one normal yellow signal (an arrowhead) in each nucleus

To reclassify the 31 cases based on genomic information, we performed FISH analysis. The total number of FISH-assessable cases was 26. All cases of AciCC that could be evaluated showed two yellow signals in each nucleus, which indicated wild-type *ETV6*. Of the 10 AciCC cases, eight were evaluable with FISH. Isolated signals indicating rearrangements were detected in patients 1–12. A single yellow signal, as well as red and green signals, were observed in each nucleus, indicating the wild-type gene and the rearranged *ETV6*, respectively. This pattern was common in SC (Fig. 1). Because of degraded nucleic acids due to prolonged storage, 5 cases were difficult to evaluate with FISH. We performed PCR/DNA sequencing analysis using the same paraffin block to diagnose SC/AciCC.

### RT-PCR and DNA sequencing analysis detected other fusion genes

DNA sequencing using cDNA was performed to further examine DNA rearrangements not detected with FISH. cDNAs from tumors of patients 1–9 generated 110-bp amplicons, consistent with the typical *ETV6* exon 5::*NTRK3* exon 15 fusion transcript in RT-PCR. In patients 10–21, nested PCR followed by RT-PCR generated the amplicon. No other fusion transcripts, such as *ETV6* exon 4::*NTRK3* exon 14 or *ETV6* exon 6::*RET* exon 12 were detected, either with primary PCR or with nested PCR. Translocations between *ETV6* exon 5 and *NTRK3* exon 15 were identified by Sanger sequencing in patients 1–21. Collectively, based on FISH and DNA sequencing, we reclassified all cases as 21 SCs and 10 AciCCs (Supplementary Table 2).

### Clinical characteristics of 31 cases

Based on genotypic and histopathological findings, we reclassified 31 cases that were originally diagnosed pathologically without genomic information as 4 SCs and 27 AciCCs. After reclassification, we found that there were 21 SCs (patient numbers: 1–21) and 10 AciCCs (patient numbers: 22–31), highlighting the difficulty of accurately diagnosing SC using only existing immunohistochemical methods. Of 21 SC cases in this study, 17 were conventionally diagnosed with detection of fusion transcripts (Table 1). Translocation of the *ETV6* gene was confirmed in all SC cases with FISH or RT-PCR followed by DNA sequence analysis. Patients with SC were significantly younger than those with AciCC (median values of 50.8 years of age for SC and 64.2 years for AciCC, *p* = 0.0424). Primary sites of SCs were the parotid gland (20 patients) and submandibular gland (1 patient). On the other hand, those of AciCC were found in the parotid gland (7 patients) and minor salivary glands (3 patients) (*p* = 0.0267).

**Table 1:** Comparison of clinical features in SC and acicc

	Total (*n* = 31)	SC (*n* = 21)	AciCC (*n* = 10)	*p*
Sex– no. (%)				0.704
male	15 (48.4)	11 (52.4)	4 (40)	
female	16 (51.6)	10 (47.6)	6 (60)	
Age				0.0424*
median	53	53	69	
IQR	45-68.5	43–58	49.25–72.25	
Primary site– no. (%)				0.0267*
Parotid	27 (87.1)	20 (95.2)	7 (70)	
Submandibular	1 (3.2)	1 (4.8)	0 (0)	
Minor salivary gland	3 (9.7)	0 (0)	3 (30)	
pT– no. (%)				0.944
pT1	13 (41.9)	9 (42.9)	4 (40)	
pT2	10 (32.3)	6 (28.6)	4 (40)	
pT3	4 (12.9)	3 (14.3)	1 (10)	
pTX	4 (12.9)	3 (14.3)	1 (10)	
pN– no. (%)				0.805
pN0	25 (80.6)	16 (76.2)	9 (90)	
pN1	2 (6.5)	2 (9.5)	0 (0)	
≧ pN2	0 (0)	0 (0)	0 (0)	
pNX	4 (12.9)	3 (14.3)	1 (10)	
First treatment– no. (%)				0.187
Only Excision	25 (80.6)	15 (71.4)	10 (100)	
Excision + RT	5 (16.1)	5 (23.8)	0 (0)	
Excision + PrT	1 (3.2)	1 (4.8)	0 (0)	
Outcome– no. (%)				0.643
Loco-regional recurrence	6 (19.4)	4 (19.0)	2 (20)	
Distant metastasis	1 (3.2)	1 (4.8)	0 (0)	
Dead of disease	1 (3.2)	0 (0)	1 (10)	

Results differed significantly in mean patient age and location of the primary site

RT: radiotherapy, PrT: proton therapy.*: *p* < 0.05

Surgery was the first treatment for all cases. Five cases of SC were treated with postoperative radiotherapy, and one was treated with postoperative proton therapy. Postoperative therapies were performed because of positive or close resection margin (2/6 cases), though without multiple lymph node metastases or extranodal invasion. Follow-up periods ranged from 1 to 143 months (average, 61 months). Prognoses in most cases were good, and only one case was fatal. Four patients with SC and two patients with AciCC relapsed locally. One of the five cases treated with radiotherapy developed local recurrence. One case treated with proton therapy has progressed without recurrence. In addition, all of these six patients are alive. Progression-free survival did not differ significantly in the SC and AciCC groups (*p* = 0.494) (Supplementary Fig. 1).

### Development of practical pathological evaluation based on IHC scoring

Immunohistochemical results are summarized in Table 2. SC tumors were diffusely positive for GATA 3 in 20/21 cases, mammaglobin in 17/21 cases, S-100 and DOG1 in 15/21 cases, and pan-Trk in 13/18 cases. No patients tested positive for α-amylase. AciCC tumors were positive for DOG1 in 9/9 cases, GATA3 in 4/8 cases, and pan-Trk in 3/8 cases. Notably, no AciCC tumors were positive for both S-100 and mammaglobin.

**Table 2:** Immunohistochemistry of SC and acicc

Results for individual antibodies	antibody (N)	SC			AciCC			*p*	OR
		positive (weak)	negative		positive (weak)		negative		
	pan-Trk (26)	13 (2)	5		3 (1)		5	0.1892	4.33
	mammaglobin (29)	17 (4)	4		0		8	0.0001**	66.11
	S-100 (30)	15 (1)	6		0		9	0.0007**	45.31
	DOG1 (30)	15 (6)	6		9 (1)		0	0.1405	0.13
	GATA3 (29)	20 (8)	1		4 (4)		4	0.0128*	20
	Ki-67 (30)	8.91% (2.3–19.4%)			5.42% (0.2–9.1%)			0.0784	
Results by intensity of positivity	antibody	negative		positive					
		- (95% CI)		weak		+		++	
	mammaglobin	33.3% (13.1–62.4)		100.0% (0-100)		100.0% (0-100)		100.0% (0-100)	
	S-100	40.0% (19.2–65.2)		100.0% (0-100)		100.0% (0-100)		100.0% (0-100)	
	GATA3	20.0% (2.72–69.1)		66.7% (37.6–86.9)		100.0% (0-100)		100.0% (0-100)	

The upper section summarizes staining results for individual antibodies. S100, mammaglobin, and GATA3 showed significant differences, while pan-Trk did not (OR: odds ratio, *: *p* < 0.05、**: *p* < 0.001). The bottom row shows the three antibodies that manifested significant differences and consistency with FISH/RT-PCR results by intensity of positivity

**Fig. 2:**
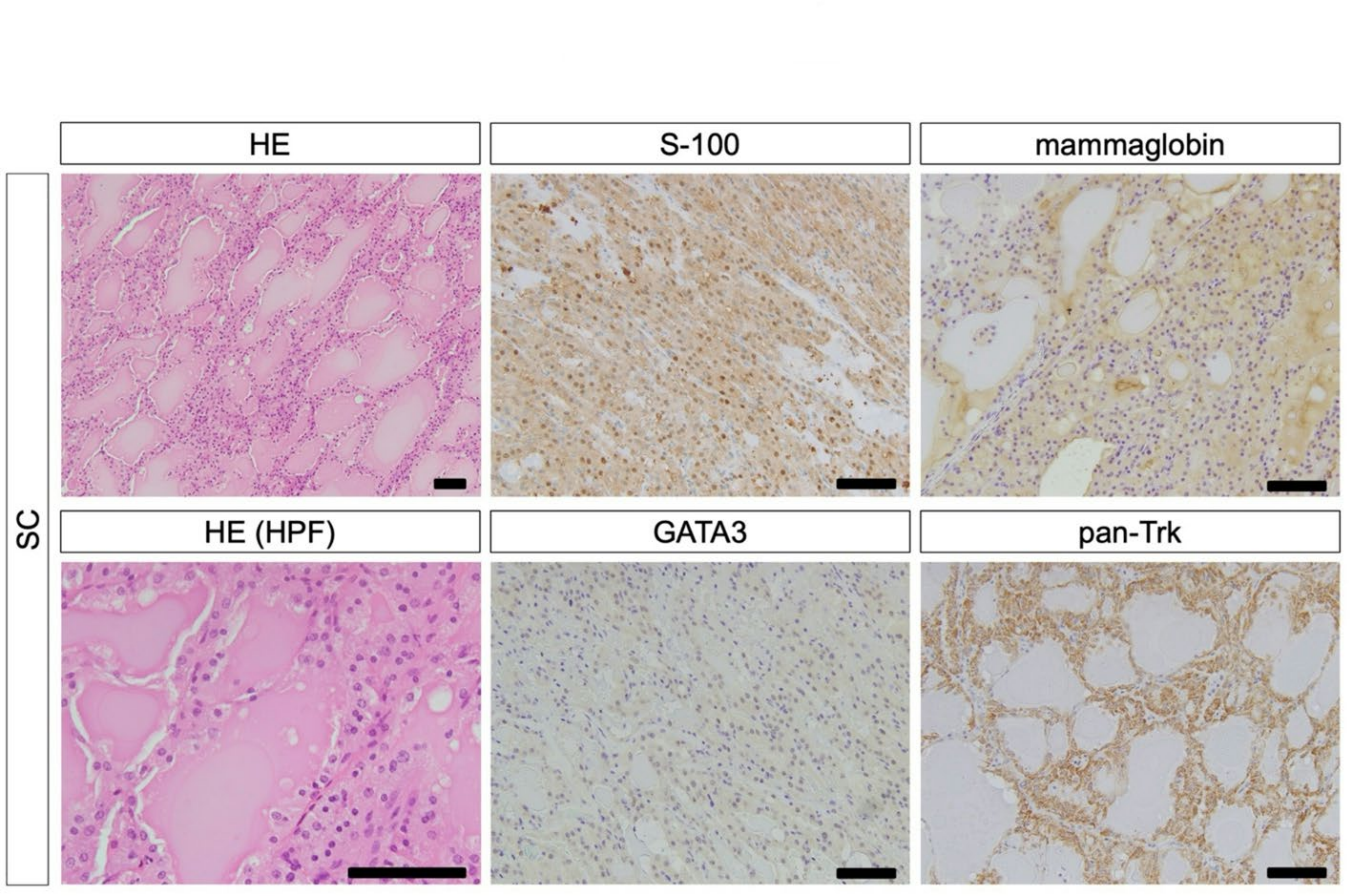
Primary histological and immunohistochemical features and cell types of SC. Papillary-cystic pattern tumor nests include abundant secretions (hematoxylin and eosin, ×100). Tumor cells had eosinophilic/clear cytoplasm (hematoxylin and eosin, ×400). Tumor cells of SC are diffusely positive for S-100 (nuclei and cytoplasms, ×200), mammaglobin (cytoplasms and secretions, ×200), GATA3 (nuclei, ×200), and pan-Trk (cytoplasms, ×200). Scale bars are 100 μm

**Fig. 3:**
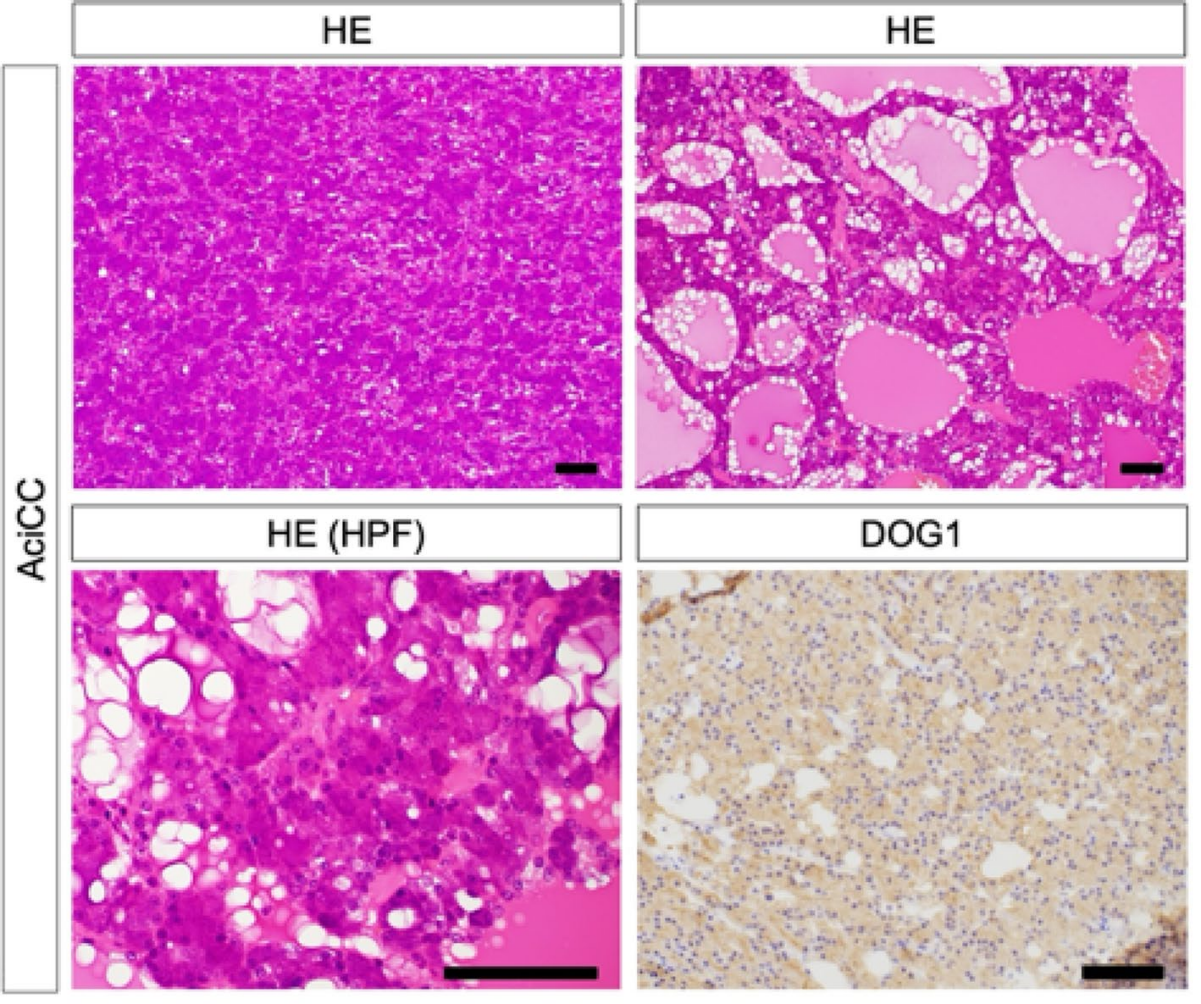
Primary histological and immunohistochemical features and cell types of AciCC. AciCC shows solid and follicular patterns (hematoxylin and eosin, ×100). Tumor cells resembled acinar cells with little atypism (hematoxylin and eosin, ×400). Tumor cells are diffusely positive for DOG1. Scale bars are 100 μm

Frequency of expression of S-100, mammaglobin, and GATA3 differed significantly between SCs and AciCCs (*p* < 0.001 for S-100 and mammaglobin, and *p* = 0.0128 for GATA3). The Ki-67 labeling index median was higher for SCs than AciCCs, but not significantly (SCs: median 8.91%, range 2.3-19.4%; and 5.42%, range 0.2-9.1%, in AciCCs; *p* = 0.0784). Pan-Trk staining results were not very definitive (*p* = 0.1892) (Figs. 2 and 3).

**Fig. 4:**
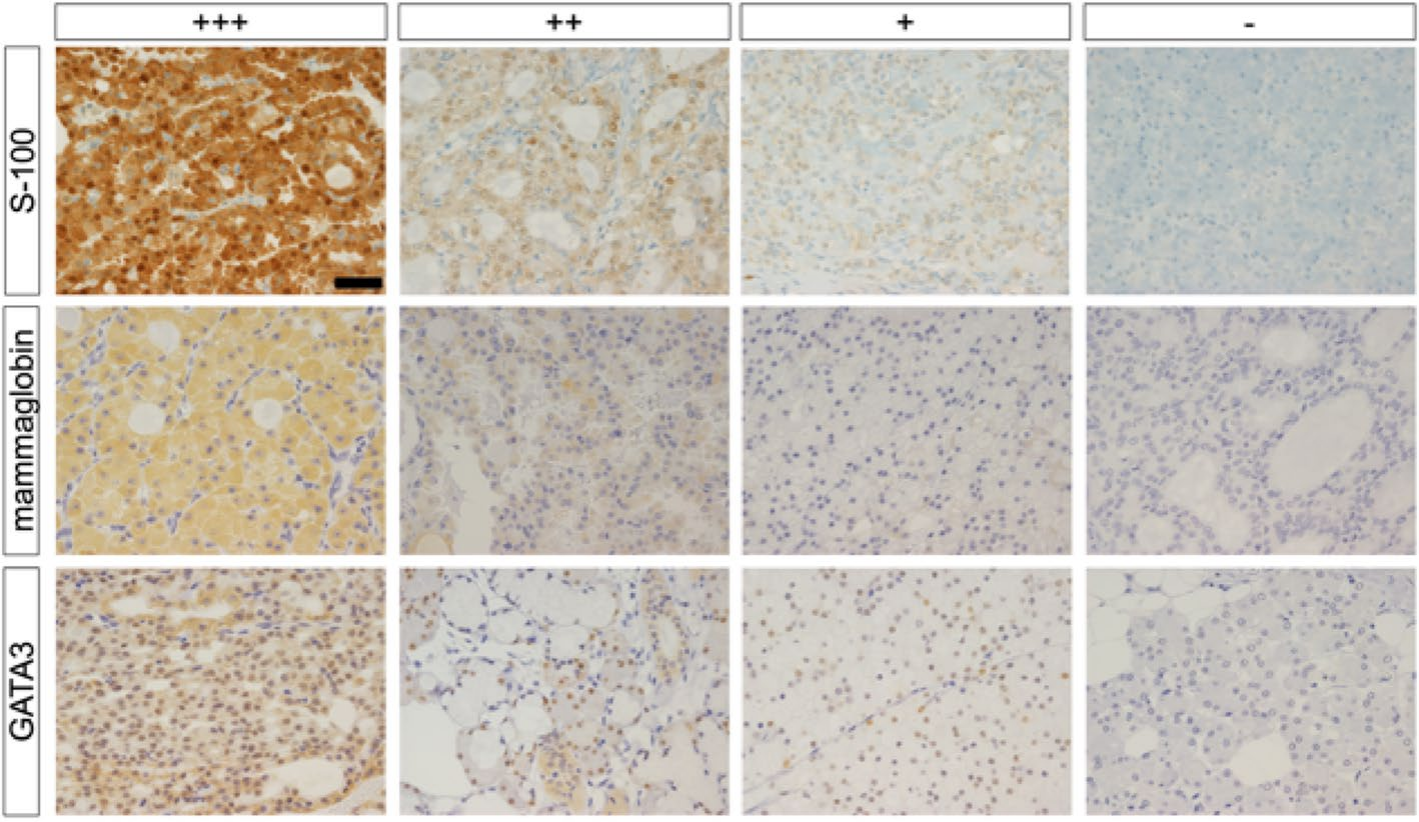
Positive/negative in real images. Microscopic images of +++, ++, + (weak), and -, in order, respectively. S-100, mammaglobin, and GATA3 staining (from top to bottom). All images were taken at ×400. Scale bars are 50 μm

Using IHC results for S-100, mammaglobin, and GATA3, which showed statistically significant differences in evaluating positivity and negativity, we tried to establish a novel pathological evaluation to distinguish SC from AciCC. Positivity was assessed at three levels (strongly positive +++, moderate positive ++, and weakly positive +), and negativity was also added to evaluate the predicted probability of SC at four levels (Fig. 4). All three antibody combinations showed almost 100% predicted probability for more than moderate to positivity, whereas there were differences in predicted probability values for weak positivity and negativity. We then calculated predicted probability in the same way to evaluate two combinations of antibodies (4 × 4 = 16 segments).

**Fig. 5:**
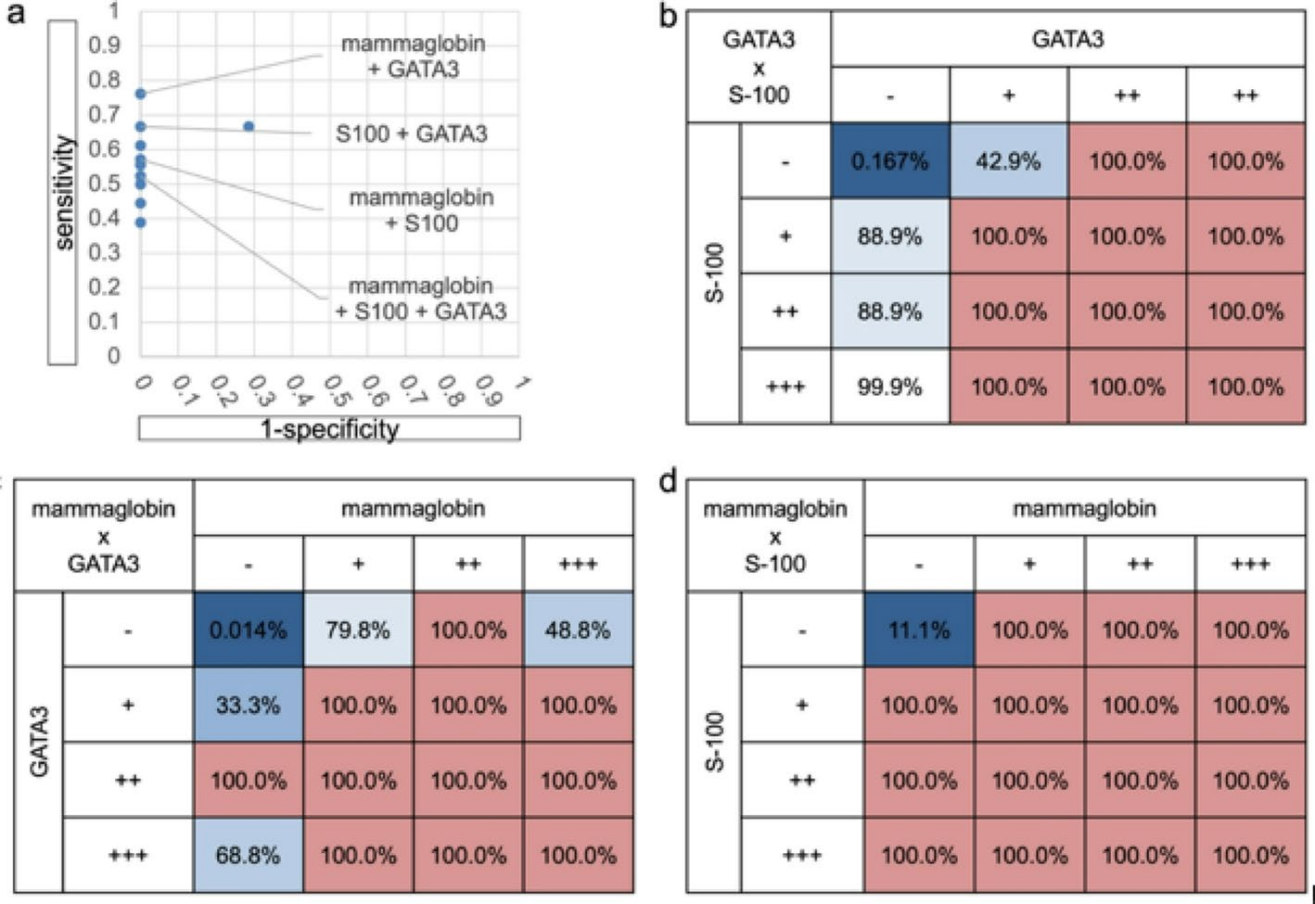
Positive predictive value (PPV) of two IHC antibodies. The grid follows the notation of the ROC curve, showing the false-positive rate on the horizontal axis and the true-positive rate on the vertical axis. Dots in the figure without antibody names are combinations with pan-Trk and combinations of three or more antibodies. The 16 contingency tables classify positives and negatives using the three antibodies, each showing significant differences

Predicted probability was more than 99% when both combinations of antibodies were weakly positive or higher. The sensitivity and specificity of the antibody combinations were as follows: sensitivity 66.7% / specificity 100% for GATA3 & S-100, 76.2% / 100% for mammaglobin & GATA3, 57.1% / 100% for mammaglobin & S-100, and 52.4% / 100% for mammaglobin & GATA3 & S-100. Notably, with S-100 and mammaglobin combined, predicted probability approximated 100%, except when both were negative, suggesting that combined S-100 and mammaglobin staining is robust for simple, reliable diagnosis of SC. Estimated probabilities by intensity of staining are shown in Fig. 5, and individual IHC results are summarized in Supplementary Table 3.

## Discussion

Immunohistochemically, SC is generally positive for S-100, mammaglobin, and CK-7, but negative for α-amylase and p63. Some cases of SC are positive for DOG1, GCDFP15, and GATA3, but the positivity rate varies [1, 2, 13, 15–20]. Recent reports indicated that anti-pan-Trk may be useful as a detection marker for *NTRK* gene fusions in SC [21, 22]. In our present study, however, pan-Trk staining was observed not only in SC, but also in AciCC to a lesser extent, suggesting pan-Trk staining is not as effective as has been previously reported. In some AciCC cases, α-amylase, DOG1, and SOX10 are positive, and mammaglobin is usually negative. This information is helpful, but not sufficiently specific to distinguish AciCC from SC. Actually, of the 10 AciCC cases in our study, nine were strongly positive for DOG1, while six of the 21 SC cases were negative and six were weakly positive; atypical results of DOG1 staining can be attributed in part to the type of anti-DOG1 antibody used and staining conditions such as dilution ratio and activation, as well as the lack of uniformity in positive criteria. Previously, attempts to differentiate SC from AciCC with multiple IHCs, rather than using only a single existing antibody, have been reported. Sharma et al. reviewed the diagnostic utility of the combination of S100, mammaglobin, SOX10, and DOG1 [23]. In this review, sensitivity and specificity using the above antibodies were also described, with sensitivity ranging from 50 to 98.4% and specificity from 50 to 86.1%.

Based on these complicated diagnostic situations, we tried to establish a practical IHC method to differentiate SC and AciCC. This study proved the usefulness of an IHC combination of S-100, mammaglobin, and GATA3. In particular, combinatorial positivity for all three of these markers reliably diagnosed SC. In addition, positivity of either S-100 or mammaglobin strongly suggests SC, except when both are negative. This method is helpful to easily distinguish SC from AciCC by IHC, reducing time and costs for genetic diagnosis during busy daily work.

*The nuclear receptor subfamily 4 group A member 3 (NR4A3)* rearrangement is often observed in AciCC [24]. The *NR4A3* gene codes *neuron-derived orphan receptor 1 (NOR-1)* protein. One study showed the usefulness of NOR1 IHC for AciCC in alcohol-fixed cytologic preparations [24]. Another indicated that nuclear NR4A2 (coding nuclear receptor related 1: Nurr1) immunostaining is helpful for AciCC lacking the *NR4A3 (NOR-1)* upregulation [25]. In addition, another recent report indicated that immunopositivity for *muscle*,* intestine*,* and stomach expression 1 (Mist1)* is a sensitive marker for serous acinar cells of AciCC [26]. Due to the emphasis on diagnosis of SC, we could not evaluate *NR4A3* or *NR4A2* in this study, except for *Mist1*. This information promotes precise diagnosis of AciCC in combination with results of this study.

Regarding genetic profiles, SC was initially reported as a tumor with an *ETV6* exon5::*NTRK3* exon15 gene fusion rearrangement [1]. Moreover, recent studies demonstrate other types of gene rearrangements, including *ETV6* exon 5::*RET* exon 12 gene rearrangement [6], dual-fusions of *ETV6* exon 5::*NTRK3* exon 15 and *ETV6* exon 2::*MAML3* exon 2 gene rearrangements [7], or *ETV6* exon 6::*RET* exon 12 and *EGFR* exon 24::*SEPT14* exon 7 gene rearrangements [10]. In addition, atypical rearrangements between exons 4 and 5 of *ETV6* and exon 14 or 15 of *NTRK3*, which are different from the classical *ETV6* exon5::*NTRK3* exon15 rearrangement, have been reported [6]. In this study, nine primary RT-PCR-positive cases were all FISH-positive. In contrast, 3 of the 12 cases with positive nested PCR were able to detect break apart by FISH, whereas 6/12 did not detect fusion and 3/12 could not be evaluated by FISH. Even in cases where break apart cannot be detected by FISH, evaluation by nested PCR may be beneficial. Although FISH and RT-PCR can detect such fusion genes to confirm diagnoses of SC, such assays cannot be used to examine all cases in busy clinical settings. On the other hand, IHC is widely used in clinical practice, suggesting that combined staining strategies may be more useful than evaluations with a single antibody. Combined approaches are expected to improve diagnostic accuracy and postoperative treatment.

Use of molecular-targeted drugs, especially entrectinib [8] and larotrectinib [9], for *NTRK* fusion-positive SCs [10] and selpercatinib [11] for *RET* fusion gene-positive SCs will prolong patient prognoses if precise diagnosis is performed. We believe that this practical, simple diagnostic method will contribute to IHC-based pre-screening before RT-PCR or FISH. This method uses a combination of two or three antibodies that are already widely employed, and is considered reasonable in terms of time and cost.

This study suffered the limitation that preservation of tumor tissue was inconsistent, since the study spanned approximately 20 years, resulting in cases that were difficult to evaluate by FISH and IHC staining. In addition, salivary gland carcinoma is a rare cancer, with an incidence of about 1.3–1.4 cases/100,000 persons/year [27, 28], and the number of SC and AciCC cases in this study was only about 30. Since there are a wide variety of combinations, especially when multiple antibodies of IHC are used, it would be desirable to accumulate cases at various institutions for more accurate evaluation and to determine ideal parameters for future studies. Second, in this study, we summarized results from multiple pathologists, indicating that there may be a need for more precise standards of judgment. In regard to RT-PCR, the sensitivity was 83.3% and the specificity was 100% in a past study on RT-PCR of glioblastoma FFPE samples [29]. FFPE samples used in this study were from 10 to 20 years ago, and it is difficult to cover all fusion genes with RT-PCR. Considering this, it is expected that the specificity should be higher, but the sensitivity should be lower in our experiment. Furthermore, the only fusion genes analyzed for SC by RT-PCR in this study were *ETV6::NTRK3* and *ETV6::RET*. Other fusion genes were not addressed. Skálová et al. investigated 49 SC cases [30], 40/49 (82%) with *ETV6::NTRK3* and 8/49 (16%) with *ETV6::RET*. One case of *ETV6::NTRK3* fusion showed a concurrent *MYB::SMR3B* fusion. In addition, one of the remaining cases had a novel *VIM::RET* fusion. Although a small number, the presence of SC cases with atypical fusion genes should be taken into account. Further molecular analyses of a large cohort of SCs may confirm the significance of our findings.

## Conclusions

In this study, we analyzed clinical, histological, immunohistochemical, and molecular characteristics of 31 cases initially diagnosed as either secretory carcinoma (SC) or acinic cell carcinoma (AciCC). Notably, more than half of the cases originally classified as AciCC were reclassified as SC, based on sequencing analyses using FISH and RT-PCR. Given the limitations of genomic testing in routine clinical practice, we sought to establish a practical immunohistochemical (IHC) diagnostic strategy for SC. Our findings demonstrate that combined use of S-100, mammaglobin, and GATA3 constitutes a highly effective IHC panel for accurate identification of SC. In particular, simultaneous positivity for all three markers was strongly indicative of SC. Moreover, the presence of either S-100 or mammaglobin alone provided supportive evidence for SC, except in cases where both were negative. In morphologically ambiguous cases, especially when genetic fusion testing is unavailable or pending, this IHC panel offers a practical and dependable tool for distinguishing SC from AciCC. Overall, although some problems such as false positives and overlap of markers with other neoplasms need to be considered, our results contribute to development of a robust and accessible diagnostic approach for SC in routine clinical settings.

## Electronic supplementary material

Below is the link to the electronic supplementary material.

Supplementary Material 1: Supplementary Table 1 Primers used in this study. Primer sequences were taken from Skálová [14]. Supplementary Table 2 Results of ETV6-FISH and RT-PCR analysis. Initial and last diagnoses after analysis are shown, as well as results of FISH and RT-PCR. Parentheses in fusion gene columns indicate results of nested PCR. Supplementary Fig. 1 Overall Survival / Progression-Free Survival. There was no significant difference between SC and AciCC in either Overall Survival (p=0.317) or Progression Free Survival (p=0.494). Supplementary Table 3 Results of IHC. Initial and last diagnoses after analysis are shown, as well as results of IHC.

## Data Availability

The datasets generated during and/or analysed during the current study are available from the corresponding author on reasonable request.

## References

[1] Skálová A, Vanecek T, Sima R, Laco J, Weinreb I, Perez-Ordonez B, et al. Mammary analogue secretory carcinoma of salivary glands, containing the ETV6-NTRK3 fusion gene: a hitherto undescribed salivary gland tumor entity. Am J Surg Pathol. 2010;34:599–608. 10.1097/pas.0b013e3181d9efcc. 20410810 10.1097/PAS.0b013e3181d9efcc

[2] Urano M, Nagao T, Miyabe S, Ishibashi K, Higuchi K, Kuroda M. Characterization of mammary analogue secretory carcinoma of the salivary gland: discrimination from its mimics by the presence of the ETV6-NTRK3 translocation and novel surrogate markers. Hum Pathol. 2015;46:94–103. 10.1016/j.humpath.2014.09.012. 25456394 10.1016/j.humpath.2014.09.012

[3] El-Naggar AKCJ, Grandis JR, Takata T, Slootweg PJ. WHO classification of head and neck tumours. 4th ed. Lyon: IARC; 2017.

[4] Skálová A, Sima R, Vanecek T, Muller S, Korabecna M, Nemcova J, et al. Acinic cell carcinoma with high-grade transformation: a report of 9 cases with immunohistochemical study and analysis of TP53 and HER-2/neu genes. Am J Surg Pathol. 2009;33:1137–45. 10.1097/pas.0b013e3181a38e1c. 19461506 10.1097/PAS.0b013e3181a38e1c

[5] Numano Y, Ogawa T, Ishikawa T, Usubuchi H, Nakanome A, Ohkoshi A, et al. Parotid secretory carcinoma with high-grade transformation. Auris Nasus Larynx. 2020;47:1043–8. 10.1016/j.anl.2019.10.003. 31679811 10.1016/j.anl.2019.10.003

[6] Skálová A, Vanecek T, Martinek P, Weinreb I, Stevens TM, Simpson RHW, et al. Molecular profiling of mammary analog secretory carcinoma revealed a subset of tumors harboring a novel ETV6-RET translocation: report of 10 cases. Am J Surg Pathol. 2018;42:234–46. 10.1097/pas.0000000000000972. 29076873 10.1097/PAS.0000000000000972

[7] Guilmette J, Dias-Santagata D, Nosé V, Lennerz JK, Sadow PM. Novel gene fusions in secretory carcinoma of the salivary glands: enlarging the ETV6 family. Hum Pathol. 2019;83:50–8. 10.1016/j.humpath.2018.08.011. 30130630 10.1016/j.humpath.2018.08.011

[8] Demetri GD, De Braud F, Drilon A, Siena S, Patel MR, Cho BC, et al. Updated integrated analysis of the efficacy and safety of entrectinib in patients with NTRK Fusion-Positive solid tumors. Clin Cancer Res. 2022;28:1302–12. 10.1158/1078-0432.ccr-21-3597. 35144967 10.1158/1078-0432.CCR-21-3597 PMC9365368

[9] Le X, Baik C, Bauman J, Gilbert J, Brose MS, Grilley-Olson JE, et al. Larotrectinib treatment for patients with TRK Fusion-Positive salivary gland cancers. Oncologist. 2022;29:e779–88. 10.1093/oncolo/oyac080. 35536733 10.1093/oncolo/oyac080 PMC11144979

[10] Drilon A, Li G, Dogan S, Gounder M, Shen R, Arcila M, et al. What hides behind the MASC: clinical response and acquired resistance to entrectinib after ETV6-NTRK3 identification in a mammary analogue secretory carcinoma (MASC). Ann Oncol. 2016;27:920–6. 10.1093/annonc/mdw042. 26884591 10.1093/annonc/mdw042 PMC4843186

[11] Subbiah V, Wolf J, Konda B, Kang H, Spira A, Weiss J, et al. Tumour-agnostic efficacy and safety of Selpercatinib in patients with RET fusion-positive solid tumours other than lung or thyroid tumours (LIBRETTO-001): a phase 1/2, open-label, basket trial. Lancet Oncol. 2022;23:1261–73. 10.1016/s1470-2045(22)00541-1. 36108661 10.1016/S1470-2045(22)00541-1 PMC11702314

[12] Pang Y, Sun L, Liu H, Ma J. Differential diagnosis and treatment of salivary secretory carcinoma and acinic cell carcinoma. Oral Oncol. 2021;119:105370. 10.1016/j.oraloncology.2021.105370. 34157553 10.1016/j.oraloncology.2021.105370

[13] Hsieh MS, Chou YH, Yeh SJ, Chang YL. Papillary-cystic pattern is characteristic in mammary analogue secretory carcinomas but is rarely observed in acinic cell carcinomas of the salivary gland. Virchows Arch. 2015;467:145–53. 10.1007/s00428-015-1786-8. 25976476 10.1007/s00428-015-1786-8

[14] Skálová A, Vanecek T, Simpson RH, Laco J, Majewska H, Baneckova M, et al. Mammary analogue secretory carcinoma of salivary glands: molecular analysis of 25 ETV6 gene rearranged tumors with lack of detection of classical ETV6-NTRK3 fusion transcript by standard RT-PCR: report of 4 cases harboring ETV6-X gene fusion. Am J Surg Pathol. 2016;40:3–13. 10.1097/pas.0000000000000537. 26492182 10.1097/PAS.0000000000000537

[15] Chiosea SI, Griffith C, Assaad A, Seethala RR. Clinicopathological characterization of mammary analogue secretory carcinoma of salivary glands. Histopathology. 2012;61. 10.1111/j.1365-2559.2012.04232.x.:387– 94. 10.1111/j.1365-2559.2012.04232.x 22372712

[16] Stevens TM, Kovalovsky AO, Velosa C, Shi Q, Dai Q, Owen RP, et al. Mammary analog secretory carcinoma, low-grade salivary duct carcinoma, and mimickers: a comparative study. Mod Pathol. 2015;28:1084–100. 10.1038/modpathol.2015.64. 26089091 10.1038/modpathol.2015.64

[17] Connor A, Perez-Ordoñez B, Shago M, Skálová A, Weinreb I. Mammary analog secretory carcinoma of salivary gland origin with the ETV6 gene rearrangement by FISH: expanded morphologic and immunohistochemical spectrum of a recently described entity. Am J Surg Pathol. 2012;36:27–34. 10.1097/pas.0b013e318231542a. 21989350 10.1097/PAS.0b013e318231542a

[18] Din NU, Fatima S, Kayani N. Mammary analogue secretory carcinoma of salivary glands: a clinicopathologic study of 11 cases. Ann Diagn Pathol. 2016;22:49–53. 10.1016/j.anndiagpath.2016.04.003. 27180060 10.1016/j.anndiagpath.2016.04.003

[19] Forner D, Bullock M, Manders D, Wallace T, Chin CJ, Johnson LB, et al. Secretory carcinoma: the Eastern Canadian experience and literature review. J Otolaryngol Head Neck Surg. 2018;47:69. 10.1186/s40463-018-0315-6. 30446016 10.1186/s40463-018-0315-6 PMC6240209

[20] Baghai F, Yazdani F, Etebarian A, Garajei A, Skalova A. Clinicopathologic and molecular characterization of mammary analogue secretory carcinoma of salivary gland origin. Pathol Res Pract. 2017;213:1112–8. 10.1016/j.prp.2017.07.017. 28781197 10.1016/j.prp.2017.07.017

[21] Hechtman JF, Benayed R, Hyman DM, Drilon A, Zehir A, Frosina D, et al. Pan-Trk immunohistochemistry is an efficient and reliable screen for the detection of NTRK fusions. Am J Surg Pathol. 2017;41:1547–51. 10.1097/pas.0000000000000911. 28719467 10.1097/PAS.0000000000000911 PMC5636652

[22] Bell D, Ferrarotto R, Liang L, Goepfert RP, Li J, Ning J, et al. Pan-Trk immunohistochemistry reliably identifies ETV6-NTRK3 fusion in secretory carcinoma of the salivary gland. Virchows Arch. 2020;476:295–305. 10.1007/s00428-019-02640-7. 31423558 10.1007/s00428-019-02640-7

[23] Sharma G, Kamboj M, Narwal A, Keerthika R, Devi A, Vijayakumar G. Diagnostic utility of expression pattern of S100/Mammaglobin/SOX10/DOG 1 immunohistochemistry in differentiation of secretory and acinic cell carcinoma: A systematic review and Meta-Analysis. Indian J Otolaryngol Head Neck Surg. 2024;76:208–18. 10.1007/s12070-023-04127-z. 38440438 10.1007/s12070-023-04127-z PMC10908910

[24] Krishnan V, Nguyen L, Shen R, Lieu D, De Peralta-Venturina M, Fan X. NOR-1 (NR4A3) immunostaining on cytologic preparations for the preoperative diagnosis of acinic cell carcinoma of the salivary gland. J Am Soc Cytopathol. 2022;11:352–8. 10.1016/j.jasc.2022.07.001. 36058827 10.1016/j.jasc.2022.07.001

[25] Haller F, Moskalev EA, Kuck S, Bieg M, Winkelmann C, Müller SK, et al. Nuclear NR4A2 (Nurr1) immunostaining is a novel marker for acinic cell carcinoma of the salivary glands lacking the classic NR4A3 (NOR-1) upregulation. Am J Surg Pathol. 2020;44:1290–2. 10.1097/pas.0000000000001494. 32341238 10.1097/PAS.0000000000001494

[26] Hsieh MS, Jeng YM, Lee YH. Mist1: a novel nuclear marker for acinic cell carcinoma of the salivary gland. Virchows Arch. 2019;475:617–24. 10.1007/s00428-019-02600-1. 31187185 10.1007/s00428-019-02600-1

[27] Tamaki T, Dong Y, Ohno Y, Sobue T, Nishimoto H, Shibata A. The burden of rare cancer in japan: application of the RARECARE definition. Cancer Epidemiol. 2014;38:490–5. 10.1016/j.canep.2014.07.014. 25155209 10.1016/j.canep.2014.07.014

[28] Institute NC, SEER Cancer Statistics Review (CSR.) 1975–2018. In: Howlader N NA, Krapcho M, Miller D, Brest A, Yu M, Ruhl J, Tatalovich Z, Mariotto A, Lewis DR, Chen HS, Feuer EJ, Cronin KA, editor.2021. https://seer.cancer.gov/csr/1975_2018/. Accessed 23 April 2025.

[29] Priesterbach-Ackley LP, van Kuik J, Tops BBJ, Lasorella A, Iavarone A, van Hecke W, et al. RT-PCR assay to detect FGFR3::TACC3 fusions in formalin-fixed, paraffin-embedded glioblastoma samples. Neurooncol Pract. 2024;11:142–9. 10.1093/nop/npad081. 38496910 10.1093/nop/npad081 PMC10940835

[30] Skálová A, Banečkova M, Thompson LDR, Ptáková N, Stevens TM, Brcic L, et al. Expanding the molecular spectrum of secretory carcinoma of salivary glands with a novel VIM-RET fusion. Am J Surg Pathol. 2020;44:1295–307. 10.1097/pas.0000000000001535. 32675658 10.1097/PAS.0000000000001535

