# Supplementary material for: Practical pathological methods for reliable diagnosis of secretory carcinomas of the salivary gland
Source: Discov Oncol. 2025 Jul 3;16:1254. doi: 10.1007/s12672-025-03072-3 (PMC12229399; doi:10.1007/s12672-025-03072-3)
Supplement: Supplementary file 1 — Supplementary Material 1: Supplementary Table 1 Primers used in this study. Primer sequences were taken from Skálová [14]. Supplementary Table 2 Results of ETV6-FISH and RT-PCR analysis. Initial and last diagnoses after analysis are shown, as well as results of FISH and RT-PCR. Parentheses in fusion gene columns indicate results of nested PCR. Supplementary Fig. 1 Overall Survival / Progression-Free Survival. There was no significant difference between SC and AciCC in either Overall Survival (p=0.317) or Progression Free Survival (p=0.494). Supplementary Table 3 Results of IHC. Initial and last diagnoses after analysis are shown, as well as results of IHC. [file 12672_2025_3072_MOESM1_ESM.pdf]

Supplementary Table 1. List of primers

Primer name	Sequences (5'–3')	Target
ETV6-ex4-F3	AGCCGGAGGTCATACTGCAT	ETV6 exon 4 inner
ETV6-ex4-F4	CATTCTTCCACCCTGGAAAC	ETV6 exon 4 outer
ETV6B	ACATCATGGTCTCTGTCTCCCCGC	ETV6 exon 5 inner
ETV6A	ACCACATCATGGTCTCTGTCTCCC	ETV6 exon 5 outer
NTRK3-ex14-R1	GTGATGCCGTGGTTGATGT	NTRK3 exon 14 inner
NTRK3 ex14-R2	AGTCATGCCAATGACCACAG	NTRK3 exon 14 outer
NTRK3B	TTCTCGCTTCAGCACGATGTCT	NTRK3 exon 15 inner
NTRK3A	CAGTTCTCGCTTCAGCACGATG	NTRK3 exon 15 outer
ETV6-Archer1-F1	CGATGGGAGGACAAAGAATC	ETV6 exon 6
RET-Archer1-R1	AACCAAGTTCTTCCGAGGGA	RET exon 12
ETV6-Archer1-F2	CAACGGACTGGCTCGACTG	ETV6 exon 6
RET-Archer1-R2	GACCACTTTTCCAAATTCGCCT	RET exon 12

Supplementary Table 2. Results of ETV6-FISH and RT-PCR

SC/AC#	initial diagnosis	last diagnosis	ETV6-FISH	*ETV6* exon5 *NTRK3* exon15	*ETV6* exon4 *NTRK3* exon14	*ETV6* exon5 *NTRK3* exon14	*ETV6* exon6 *RET* exon12
SC-1	SC	SC	break apart	+ (+)	- (-)	- (-)	- (-)
SC-2	AciCC	SC	break apart	+ (+)	- (-)	- (-)	- (-)
SC-3	AciCC	SC	break apart	+ (+)	- (-)	- (-)	- (-)
SC-4	AciCC	SC	break apart	+ (+)	- (-)	- (-)	- (-)
SC-5	AciCC	SC	break apart	+ (+)	- (-)	- (-)	- (-)
SC-6	AciCC	SC	break apart	+ (+)	- (-)	- (-)	- (-)
SC-7	AciCC	SC	break apart	+ (+)	- (-)	- (-)	- (-)
SC-8	AciCC	SC	break apart	+ (+)	- (-)	- (-)	- (-)
SC-9	AciCC	SC	break apart	+ (+)	- (-)	- (-)	- (-)
SC-10	SC	SC	break apart	- (+)	- (-)	- (-)	- (-)
SC-11	AciCC	SC	N/A	- (+)	- (-)	- (-)	- (-)
SC-12	AciCC	SC	normal	- (+)	- (-)	- (-)	- (-)
SC-13	AciCC	SC	N/A	- (+)	- (-)	- (-)	- (-)
SC-14	AciCC	SC	normal	- (+)	- (-)	- (-)	- (-)
SC-15	AciCC	SC	normal	- (+)	- (-)	- (-)	- (-)
SC-16	SC	SC	break apart	- (+)	- (-)	- (-)	- (-)
SC-17	AciCC	SC	normal	- (+)	- (-)	- (-)	- (-)
SC-18	AciCC	SC	normal	- (+)	- (-)	- (-)	- (-)
SC-19	AciCC	SC	break apart	- (+)	- (-)	- (-)	- (-)
SC-20	AciCC	SC	normal	- (+)	- (-)	- (-)	- (-)
SC-21	SC	SC	N/A	- (+)	- (-)	- (-)	- (-)
AC-1	AciCC	AciCC	normal	- (-)	- (-)	- (-)	- (-)
AC-2	AciCC	AciCC	normal	- (-)	- (-)	- (-)	- (-)
AC-3	AciCC	AciCC	normal	- (-)	- (-)	- (-)	- (-)
AC-4	AciCC	AciCC	normal	- (-)	- (-)	- (-)	- (-)
AC-5	AciCC	AciCC	N/A	- (-)	- (-)	- (-)	- (-)
AC-6	AciCC	AciCC	normal	N/A	N/A	N/A	N/A
AC-7	AciCC	AciCC	N/A	N/A	N/A	N/A	N/A
AC-8	AciCC	AciCC	normal	N/A	N/A	N/A	N/A
AC-9	AciCC	AciCC	normal	N/A	N/A	N/A	N/A
AC-10	AciCC	AciCC	normal	- (-)	- (-)	- (-)	- (-)

Supplementary Figure 1. Overall Survival / Progression-Free Survival

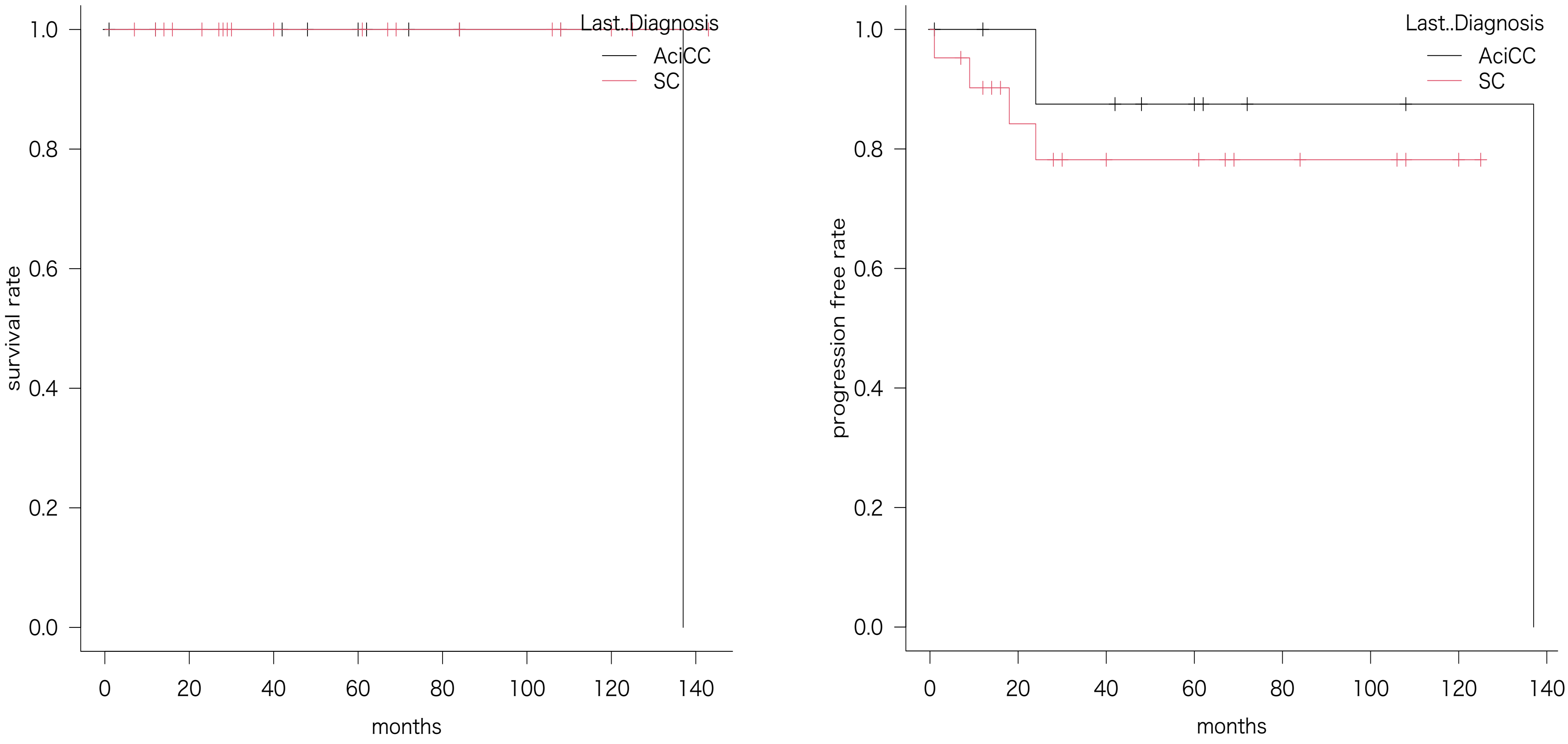

Supplementary Table 3. Results of IHC

SC/AC#	initial diagnosis	last diagnosis	pan-Trk	mammaglobin	S-100	GATA3	DOG1	Ki-67
SC-1	SC	SC	-	-	+++	++	+	11.1%
SC-2	AciCC	SC	++	+	+++	++	+++	8.5%
SC-3	AciCC	SC	+	+++	+++	++	+	9.2%
SC-4	AciCC	SC	+++	++	+++	++	+	10.3%
SC-5	AciCC	SC	-	+	+++	++	++	2.3%
SC-6	AciCC	SC	N/A	++	+++	++	+	11.7%
SC-7	AciCC	SC	+++	++	+++	++	+++	12.6%
SC-8	AciCC	SC	N/A	++	+++	++	+	8.0%
SC-9	AciCC	SC	N/A	++	+++	++	++	19.4%
SC-10	SC	SC	+++	++	+++	+	-	19.0%
SC-11	AciCC	SC	++	++	+++	-	-	2.5%
SC-12	AciCC	SC	+++	+++	-	+++	+++	2.5%
SC-13	AciCC	SC	++	++	-	+	+	6.0%
SC-14	AciCC	SC	++	++	-	+++	+++	12.5%
SC-15	AciCC	SC	-	+	-	+	+++	7.5%
SC-16	SC	SC	++	++	+++	+	-	5.5%
SC-17	AciCC	SC	+	-	+	+	+++	4.0%
SC-18	AciCC	SC	-	+	-	+	++	2.5%
SC-19	AciCC	SC	+++	-	++	+	-	16.5%
SC-20	AciCC	SC	-	-	-	++	-	12.5%
SC-21	SC	SC	+++	++	+++	+	-	3.0%
AC-1	AciCC	AciCC	+	-	-	+	+++	1.9%
AC-2	AciCC	AciCC	-	-	-	+	+++	7.0%
AC-3	AciCC	AciCC	-	-	-	-	+++	4.6%
AC-4	AciCC	AciCC	+++	-	-	+	+++	7.8%
AC-5	AciCC	AciCC	++	-	-	-	+++	3.7%
AC-6	AciCC	AciCC	N/A	N/A	-	N/A	+++	7.0%
AC-7	AciCC	AciCC	-	-	-	+	+++	0.2%
AC-8	AciCC	AciCC	-	-	-	-	+++	9.1%
AC-9	AciCC	AciCC	N/A	N/A	N/A	N/A	N/A	N/A
AC-10	AciCC	AciCC	-	-	-	-	+	7.5%
